# Hierarchical Wrinkles for Tunable Strain Sensing Based on Programmable, Anisotropic, and Patterned Graphene Hybrids

**DOI:** 10.3390/polym14142800

**Published:** 2022-07-09

**Authors:** Zengyong Chu, Guochen Li, Xiaofeng Gong, Zhenkai Zhao, Yinlong Tan, Zhenhua Jiang

**Affiliations:** College of Liberal Arts and Science, National University of Defense Technology, Changsha 410073, China; lgc_nudt@126.com (G.L.); 17755807283@163.com (X.G.); zhekai_zhao98@163.com (Z.Z.); tanyinlong15@nudt.edu.cn (Y.T.); jiangzhenhua@nudt.edu.cn (Z.J.)

**Keywords:** reduced graphene oxide (RGO), wrinkle, strain sensors, electronic materials

## Abstract

Flexible, stretchable, wearable, and stable electronic materials are widely studied, owing to their applications in wearable devices and the Internet of Things. Because of the demands for both strain-insensitive resistors and high gauge factor (GF) strain-sensitive materials, anisotropic strain sensitivity has been an important aspect of electronic materials. In addition, the materials should have adjustable strain sensitivities. In this work, such properties are demonstrated in reduced graphene oxide (RGO) with hierarchical oriented wrinkle microstructures, generated using the two-step shrinkage of a rubber substrate. The GF values range from 0.15 to 28.32 at 100% strain. For device demonstrations, macrostructure patterns are designed to prepare patterned wrinkling graphene at rubber substrate (PWG@R). Serpentiform curves can be used for the constant-value resistor, combined with the first-grade wrinkles. Strip lines can increase the strain-sensing property, along with the second-grade wrinkles. The patterned sensor exhibits improved GF values range from 0.05 to 49.5. The assembled sensor shows an excellent stability (>99% retention after 600 cycles) with a high GF (49.5). It can monitor the vital signs of the throat and wrist and sense large motions of fingers. Thus, PWG@R-based strain sensors have great potential in various health or motion monitoring fields.

## 1. Introduction

In recent years, the design and assembly of flexible [1,2] and wearable [3,4] electronic devices have attracted research interest with growing applications in robot perception [5], human movement detection [3,6], and health monitoring [7,8]. Moreover, highly integrated flexible sensors are increasingly becoming an indispensable part of the architecture of the Internet of Things [9]. However, these applications require flexible electronic devices to have excellent sensitivity, repeatability, and stability [10,11,12]. Flexible sensors are prepared by combining conductive materials with flexible substrates [8]. To ensure good tensile properties for electronic devices, the conductive materials need to have good physical and mechanical properties, and special structures are designed to improve these [13].

A strain sensor is a simple and widely used electrical device, which converts mechanical deformation into electrical signals [8]. The type of the strain sensor can be classified into piezoelectric [1], piezoresistive [7,9,12], and resistive [10,13]. To ensure flexibility, researchers compose flexible polymer substrates (e.g., polydimethylsiloxane (PDMS) [14], rubber [15], thermoplastic polyurethane [16,17], and hydrogel [18,19,20,21]) with conductive nanomaterials (e.g., graphene [22,23], carbon nanotubes (CNT) [24,25], and MXene [16,26]). The recombination mode can be classified as a filling type [27], sandwich type [24], or adsorption type [28]. The response of flexible electronic materials to strain is known to depend on the material properties, and strain sensors require maximum deformation response and sensitivity.

Graphene, with a two-dimensional honeycomb lattice structure, has excellent electrical [29,30] and sensing [31] properties suitable for flexible electronic devices [32]. Shi et al. [28] prepared graphene oxide (GO)-coated polyurethane composite foam with different pore sizes using the self-assembly method. The foam was flexible, could withstand up to 90% deformation, and reduced resistance by up to three orders of magnitude. Meanwhile, Yao et al. [22] reported a breathable and wearable electronic textile strain sensor based on a reduced GO (rGO)/CNTS hybrid material with high sensitivity and wear resistance and a gauge factor (GF) of up to 34.69.

Wrinkles are generally considered as an excellent way for imparting flexibility and stability [33]. For example, skin wrinkles at joints allow us to move flexibly without tearing the skin. The size and shape of wrinkles in materials can be controlled by different shrinkage rates, and hierarchical [34] and multi-orientation folds can be constructed to achieve different effects. Tan et al. [35] developed a method for forming hierarchical wrinkled graphene on balloons, which can be used as flexible actuators. Song et al. [15] introduced wrinkles on a substrate pre-stretching using different methods and studied the hydrophobic properties and strain performance. Jia et al. [36] reported on the preparation of graphene films with periodic and parallel wrinkles by using a pre-stretched PDMS substrate. They controlled the width of the folds in graphene by changing the strain of the pre-stretched substrate. This texturing method can be extended to other two-dimensional materials, and it has potential applications in flexible electronics and actuators.

However, electronic materials which are used as resistors should be strain-resistant; thus, resistance changes should be minimized. Louis et al. [37] demonstrated photolithography on different structures on PDMS so that the liquid metal filled in them forms a conductive pathway, which is a novel method for multi-application electronic materials. Through reasonable structural design, the desired strain response can be obtained. However, its preparation process is complicated and impractical, and the strain can only reach 50%.

Therefore, a design strategy for electronic materials with an adjustable strain-sensing response was introduced based on wrinkles and pattern. By controlling the two release directions, two-grade hierarchical wrinkles are obtained, thus producing anisotropy. Owing to the enhancement of first-grade wrinkles, additional conductive paths are present in the second-grade wrinkles, which were easily separated in strain and reconnected upon release. To obtain the desired response, the structures were designed at both macro- and micro-level by drawing designed patterns on pre-stretched rubber substrates with GO and then releasing the pre-stretched strain. Then, hydrazine vapor reduction was used to transfer GO to rGO. Because of the adjustable strain-sensing property resulting in the programmable preparation process, the prepared PWG@R is a novel electronic material that can be used as a sensitive strain sensor or a constant resistor.

## 2. Materials and Methods

**Materials:** Flake graphite, sulfuric acid, sodium nitrate, hydrochloric acid, hydrogen peroxide, anhydrous ethanol, and hydrazine hydrate were purchased from Sinopharm, Beijing, China. All reagents were used directly without further purification. The natural latex rubber film (10 cm × 10 cm × 0.1 cm) was provided by Shenzhen Jitian Chemical Co., Ltd., Shenzhen, China.

**Preparation of GO–ethanol solutions:** The modified Hummers method [38] was used to prepare a concentrated solution of GO. Then, 1 mL GO was dried for the concentrated GO solution. Ethanol was added to dilute the solution to the desired concentration.

**Preparation of PWG@R:** The natural latex rubber surface was washed with ethanol for 3 times. The rubber was stretched and fixed, and a metal mask was used to impart a pattern on the substrate. GO with a designed concentration (1~5 mg/mL) was then sprayed on the masked substrate. After the evaporation of ethanol, a coating of patterned GO film was formed on the rubber. The release was controlled in one direction at a time. Finally, the PWGO@R was reduced in hydrazine vapor at 90 °C for 4 h to obtain PWG@R.

**Characterization:** The microstructure and morphology of the samples were observed via scanning electron microscopy at 10 kV (SEM; Hitachi S-4200, Hitachi, Japan). Fourier transform infrared spectroscopy (FT-IR; Thermo Scientific Nicolet iS5, Thermo Fisher Scientific, Waltham, MA, USA) was used with an attenuated total reflectance attachment. Raman spectroscopy was performed using a HORIBA Scientific (Kyoto, Japan) LabRAM HR Evolution system with a 532 nm laser as the excitation source. High-resolution X-ray photoelectron spectroscopy (XPS; Thermo Fisher Scientific, Waltham, MA, USA) analysis was also performed using an EscaLab Xi+ instrument equipped with monochromatic Al Kα radiation (1486.6 eV), where an energy of 100 eV or 30 eV was used for the survey or high-resolution scans, respectively.

**Mechanical and strain-sensing testing:** The mechanical properties of the electronic materials were tested using an electronic mechanic testing machine (STD5000) at a compression rate of 2 mm min^−1^. The strain-sensing tests were performed under the same conditions, and the direct current (DC) and electrical resistance were measured using a multimeter (Keithley 2750). A four-probe method was used to measure the square resistance values of samples. All the mechanical and strain-sensing testing were carried out at room temperature. No additional stabilization process was needed before the measurement. Gauge factor (GF) was calculated according to Equation (1).

(1) $$\mathrm{GF}=\frac{\Delta R}{R_{0}\varepsilon }=\frac{\;R\prime -R_{0}}{R_{0}\varepsilon }.$$

## 3. Results

### 3.1. Microstructure Evolutions of PWG@R

**Figure 1:**
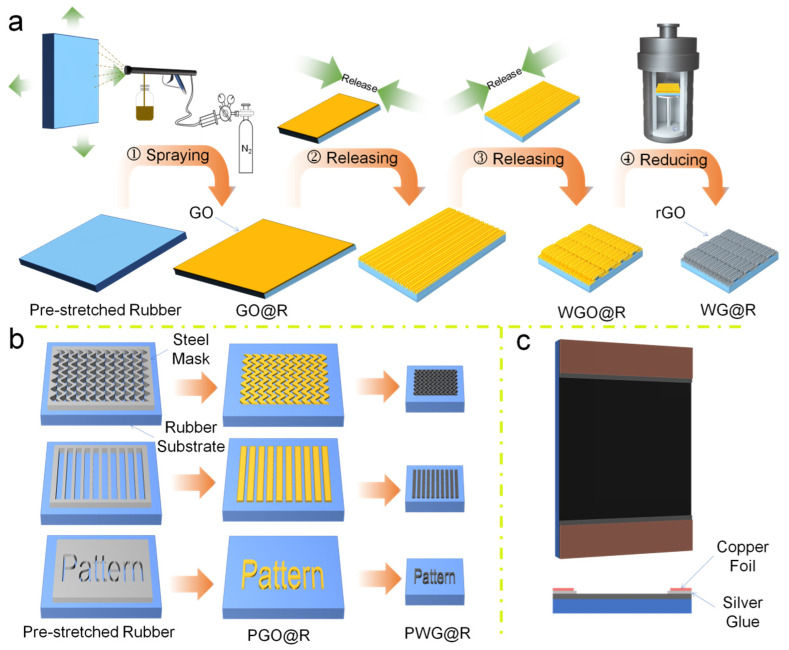
The schematic illustration of the fabrication process. (**a**) Fabrications of WG@R, (**b**) patterning of various PWG@Rs, and (**c**) the assembly of the PWG@R-based strain sensor.

Figure 1 shows the fabrication process of the (patterned) wrinkled graphene film on the rubber substrate and the assembled strain sensors. The four-step fabrication process is shown in Figure 1a. First, the GO solution was sprayed on the pre-stretched rubber substrate. Then, it was air-dried to form the graphene oxide film (GO@R). The prepared GO@R was then released in all directions sequentially to produce the GO film with the first- and the second-degree wrinkles (WGO@R). Finally, hydrazine vapor reduction was performed at 90 °C in a hydrothermal reactor to reduce the WGO@R to WG@R. The pattern was implemented with a patterned-carved steel mask. Owing to the customized and diverse steel mask designs, the prepared PWG@Rs were programmable, as shown in Figure 1b. To test the strain sensitivity of the film, PWG@R was assembled as a strain sensor using a copper foil and silver glue (Figure 1c).

**Figure 2:**
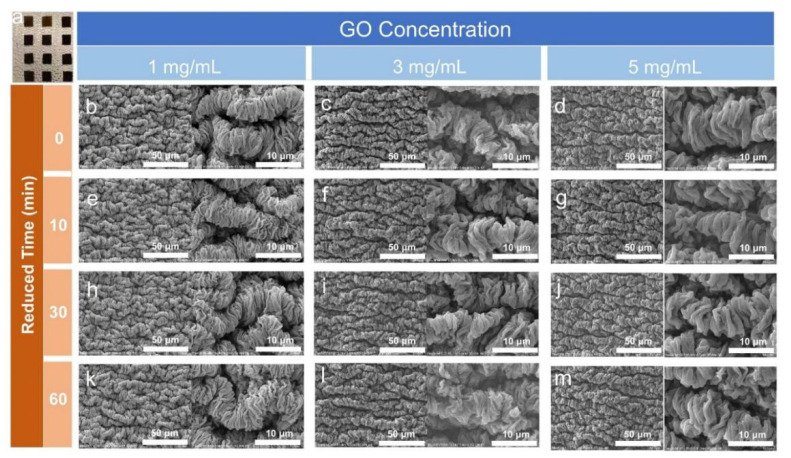
Optical images of (**a**) WG@R and SEM images of WG@R prepared with different concentrations and different reduction times. (**b**) WGO@R-1, (**c**) WGO@R-2, (**d**) WGO@R-5, (**e**) WG@R-1-10, (**f**) WG@R-2-10, (**g**)WG@R-5-10, (**h**) WG@R-1-30, (**i**)WG@R-2-30, (**j**) WG@R-5-30, (**k**) WG@R-1-60, (**l**) WG@R-2-60, and (**m**) WG@R-5-60.

To explore the influence of GO concentration and reduction time, a series of samples were prepared by varying GO–ethanol solutions (1, 3, 5 mg mL^−1^) and varying hydrazine vapor reduction times (0 min, 10 min, 30 min, and 60 min). The sample name WG@R-1-10 refers to the sample with GO concentration of 1 mg mL^−1^ and hydrazine vapor reduction time of 10 min. The optical photo in Figure 2a shows the deepening color of the sample with the increase of concentration and reduction time. The width and depth of the wrinkles were greatly affected by the concentration of GO. The thicknesses of the primary wrinkles at the concentration of 1 mg mL^−1^ and 5 mg mL^−1^ are approximately 30 nm and 200 nm, respectively. The lower concentration is conducive to the formation of denser wrinkles. With the increase of reduction time, the samples with the same concentration showed no difference, but the long reduction time (more than 120 min) caused damage to the graphene folds, which might be due to the destruction of the folded graphene structure during the reduction of oxygen-containing groups.

**Figure 3:**
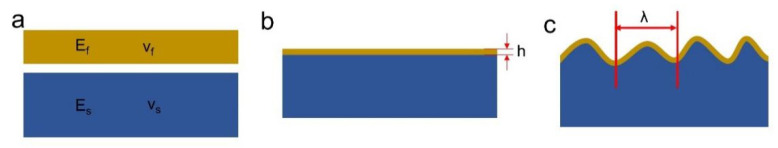
Schematic diagram of the plane wrinkling. (**a**) A planar double-layer hard and soft system, (**b**) the system in stretching mode and (**c**) the system in released mode.

Furthermore, the theory of plane wrinkling was studied to understand the influence of wrinkled morphology. For the simplest planar double-layer hard and soft systems, the thickness of the substrate is much greater than the thickness of the film. Thus, the influence of the substrate thickness is not considered. The elastic modulus of the surface film is much larger than that of the substrate. Therefore, stress induces the surface instability of the double-layer structure, and sinusoidal folds are generated owing to mismatched strains, as shown in Figure 3.

The wavelength of wrinkles caused by surface instability is related to the elastic modulus, Poisson’s ratio, and the coating thickness of the bilayer structure, which can be deduced according to its physical process [27] as follows:

(2) λ=2πh[(1−νs2)Ef(1−νf2)Es]13,

where λ is the wavelength of plane wrinkling, as shown in Figure 3c; *h* is the thickness of coating, as shown in Figure 3b; $E_{f},\;E_{s}$, and $\nu_{f},$ $\nu_{s}$ represent the elastic modulus and Poisson’s ratio of the coating and the substrate, respectively. The coating thickness then becomes the decisive factor of wavelength in surface wrinkling when both coating and substrate are fixed. The larger the thickness, the wider the formed folds, which is consistent with the SEM images in Figure 2.

### 3.2. Physical and chemical properties of WG@R

**Figure 4:**
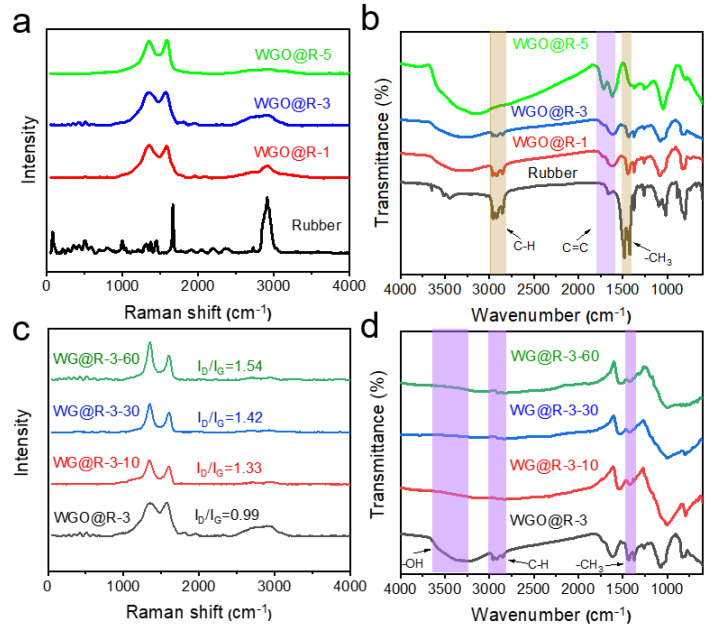
Chemical microstructures of WGO@R and WG@R. (**a**) Raman and (**b**) FT-IR spectra of rubber, WGO@R-1, WGO@R-2, and WGO@R-5; (**c**) Raman and (**d**) FT-IR spectra of WGO@R-2, WG@R-2-10, WG@R-2-30, and WG@R-2-60.

The infrared and Raman spectra of WG@R samples prepared with different GO concentrations were explored to characterize the spectral changes before and after coating. The Raman spectra (Figure 4a) show that all samples have two peaks, the D band and the G band, located at 1350 cm^−1^ and 1580 cm^−1^, respectively. The G band represents the in-plane stretching vibration of C sp^2^ (graphitized carbon), while the D band represents that of defects in the carbon lattice [39]. The peak around 2900 cm^−1^ belongs to the C–H bond in the rubber substrate, and its strength decreases as the GO solution concentration increases. In contrast, the characteristic D and G peaks of GO increase in intensity, which indicates that its adhesion thickness on the surface also increases with GO concentration, and its covering and shielding effect on the rubber substrate becomes stronger.

The infrared spectra in Figure 4b also demonstrate this. With the increase of GO concentration, the tensile vibration peaks of the C–H bond at approximately 2900 cm^−1^ and the –CH_3_ bond at approximately 1450 cm^−1^ gradually decrease, indicating that the rubber substrate is covered. At the same time, the intensity of the hydroxyl and carbon–carbon double bond vibration peaks increased, indicating an increase in the thickness of GO.

As a preliminary study of the samples prepared using 3 mg mL^−1^ GO, the GO functional group changes during the reduction process. With the increase of the reduction process, the increase of I_D_/I_G_ value can be observed from the Raman spectra (Figure 4c), which gradually increases from 0.99 to 1.54. This is owing to the formation of more defective or amorphous carbon after the removal of oxygen-containing groups. The reduction process can also be observed in the infrared spectra in Figure 4d, especially the disappearance of hydroxyl stretching vibration peak near 3400 cm^−1^, indicating a good reduction effect of hydrazine steam. Moreover, a longer reduction time causes the disappearance of the vibration peak of the C–H bond in the methyl group belonging to rubber, which may be caused by the higher infrared absorption of the reduced GO, which further covers the characteristic peak of the rubber.

**Figure 5:**
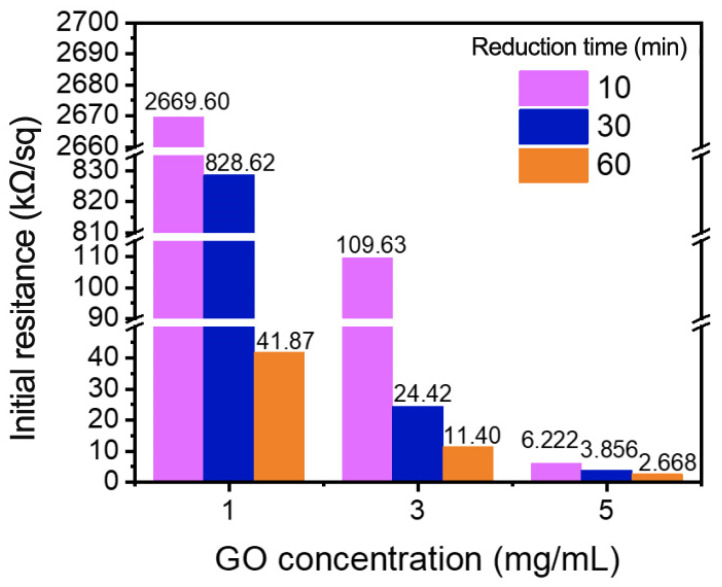
Initial resistance of WG@R with different concentrations and reduction times.

The initial resistivity is a critical parameter for the use of graded wrinkled graphene as a flexible sensing material, and so, it is essential to explore the effects of GO concentration and reduction time on the conductivity of the sample. A four-probe method was used to measure the square resistance values of samples with different concentrations and reduction times. The data are presented in Figure 5.

At the same GO concentrations, longer reduction times lead to lower square resistance values, indicating that the hydrazine steam has a good reduction effect on GO. When comparing the samples with the same reduction time, the square resistance decreases with the increase of the GO concentration, which is attributed to the increase of the graphene coating thickness. This has a positive effect on the overall conductivity of the sample. Thus, by adjusting the GO concentration and the reduction time, graded wrinkling graphene with square resistances in the range of 2.668–2669.6 kΩ/sq was prepared. The large resistance span is suitable for different applications.

**Figure 6:**
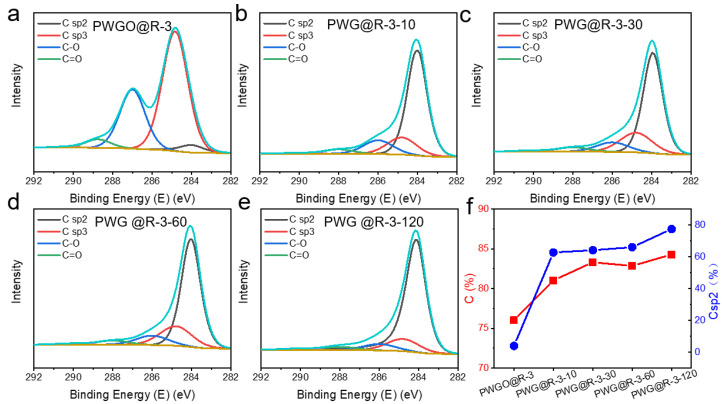
High-resolution XPS C 1s spectra of (**a**) WGO@R-2, (**b**) WG@R-2-10, (**c**) WG@R-2-30, (**d**) WG@R-2-60, and (**e**) WG@R-2-120. (**f**) The trend line of the content that is C in all atoms, and the content that is C in sp^2^ in all C atoms.

To further characterize the chemical reactions in the reduction process, the X-ray photoelectron spectra of the samples under different reduction times were tested with WGO@R-3 as the initial state, as shown in Figure 6.

As shown in Figure 6a–e, initial WGO@R-3 shows strong C–O and C=O peaks (located at 287.0 eV and 288.8 eV, respectively) and carbon atoms mainly exist in the form of sp^3^ hybridization (284.9 eV). However, in WG@R-3-10 and subsequent samples, the sp^3^ hybrid carbon atoms changed to sp^2^ state (184.1 eV), and the oxygen atom content decreased gradually. These changes are indicators of effective GO reduction. Figure 6f summarizes the changes of carbon atoms in the reduction process. The square red line indicates that the ratio of carbon atoms to total atoms increases with the increase of reduction time, because oxygen-containing functional groups are removed in the reduction process. The blue dots represent the increasing proportion of sp^2^ hybridized carbon atoms to the total carbon atoms, which means that the carbon atoms are graphitized. This is an important indicator of GO reduction.

The above analysis indicates that a chemical change occurs during the reduction process, converting GO to rGO, which provides the basis for the electrical conductivity of the WG@R sample.

**Figure 7:**
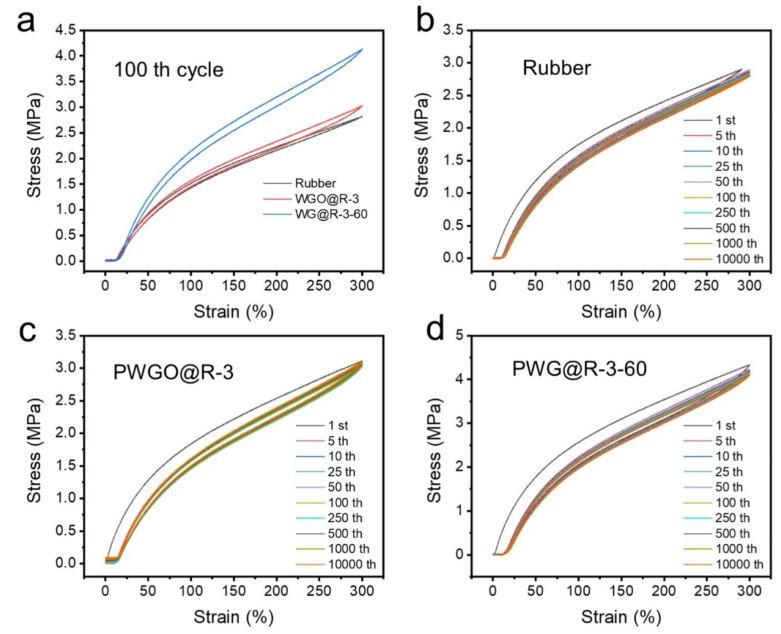
(**a**) Comparison of the mechanical cyclic properties of (**b**) rubber, (**c**) PWGO@R-3, and (**d**) PWG@R-3-60.

The mechanical properties of strain-sensing materials are also important. Mechanical properties of the rubber substrate, rubber with hierarchical wrinkled GO and reduced samples were tested. The effect of coating on the mechanical properties was qualitatively analyzed with 3 mg mL^−1^ GO concentration and 60 min reduction time as representatives, as shown in Figure 7. Rubber, WGO@R-3, and WG@R-3-60 samples were tested at a speed of 2 mm min^−1^ and their stress–strain curves were compared, as shown in Figure 7a. The GO coating exhibited minimal effect on the mechanical properties of the rubber substrate, but the reduced samples have higher tensile strength, which is enhanced by the newly formed chemical bonds during the hydrazine vapor reduction process. Figure 7d shows the fatigue resistance of the three materials, indicating that graded folded graphene on a rubber substrate is mechanically stable and can withstand tens of thousands of tensile cycles.

### 3.3. Strain-Sensing Properties of WG@R and PWG@R

The GFs of the samples were analyzed to explore the strain-sensing properties of anisotropic graded wrinkled graphene and to discuss the effects of raw material concentration and reduction time. On this basis, the macroscopic pattern was designed to further improve the GF.

**Figure 8:**
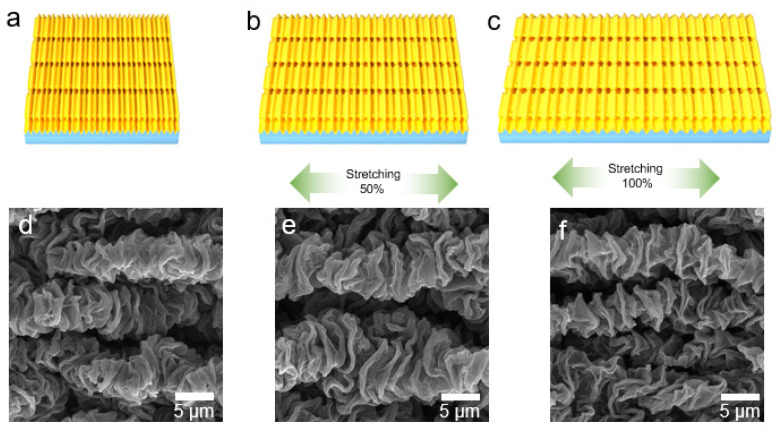
Diagrams of the stretching at (**a**) initial, (**b**) 50% strain, and (**c**) 100% strain. SEM images of stretching at (**d**) initial, (**e**) 50% strain, and (**f**) 100% strain.

Figure 8 shows the microstructure changes along the direction of primary wrinkles during stretching. Figure 8a–c shows the schematic of morphology changes generated by stretching in this direction. Figure 8d–f shows the corresponding SEM images. When tensile strain was applied to WG@R-3-60, the primary wrinkles in the microstructure become wider and the distance between folds increased. This process did not separate the original contact, and no cracks or damages were formed.

Both the schematic diagram and the electron micrograph illustrate the tensile reversibility along the primary wrinkle direction. This process is highly reversible, which is attributed to the high elasticity of the rubber substrate and the stable structure of the wrinkled graphene film. Therefore, under 100% strain, during the tensile process, there was minimal change in the conductive path of the whole material and no damage and microcracks were observed on the material.

**Figure 9:**
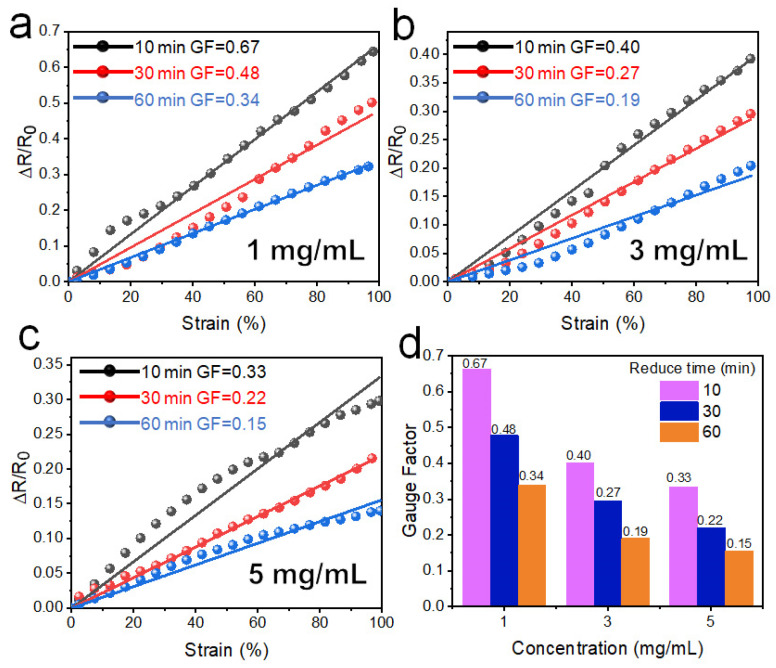
Strain-sensing curves for different reduction times at concentration of (**a**) 1 mg mL^−1^, (**b**) 3 mg mL^−1^, and (**c**) 5 mg mL^−1^. (**d**) Statistical bar chart of GF for various concentrations and reduction times.

Figure 9a–c shows the relative resistance change as a function of the strain, with different reduction times and GO concentrations. The GF is in the range of 0.15–0.67. At the same GO concentrations, GF decreases with the increase of reduction time; at the same reduction time, GF decreases with the increase of the concentration of GO. So, the thick wrinkled graphene layer leads to the lower GF. The thick layer is not prone to the occurrence of tiny cracks in the tensile process, resulting in little change of resistance.

Figure 9d shows the variation of GFs and demonstrates that high concentrations and long reduction times lead to small GFs, and thus, lower strain sensitivity. The square resistance values showed a similar trend: small resistance values lead to smaller GF. Thus, thicker films have better adhesion during longer reduction time and show less structural change during the tensile process. Typically, PWG@R-5-60 has a resistance change of <15% at 100% strain, which has a potential for strain-insensitive resistance in flexible devices.

**Figure 10:**
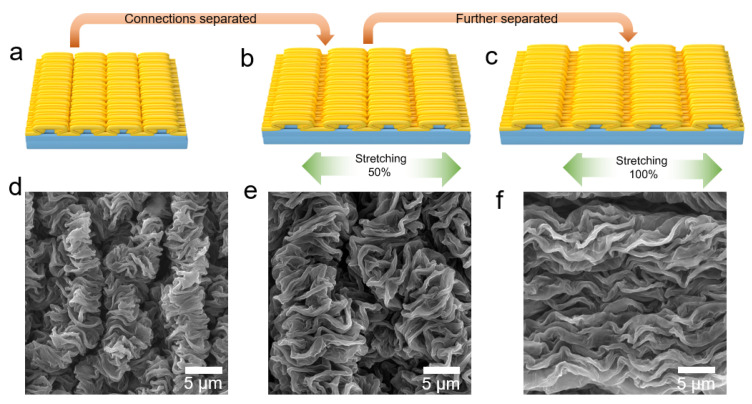
Diagrams of the stretching at (**a**) initial, (**b**) 50% strain, (**c**) 100% strain; SEM images of the stretching at (**d**) initial, (**e**) 50% strain, (**f**) 100% strain.

The schematic diagram in Figure 10a–c demonstrates the separation of the connection of the fold surface during the stretching process in the direction of the second-order fold. The corresponding SEM images in Figure 10d–f also supports this model, and the separation of the original connection generated in the stretching process can be observed.

**Figure 11:**
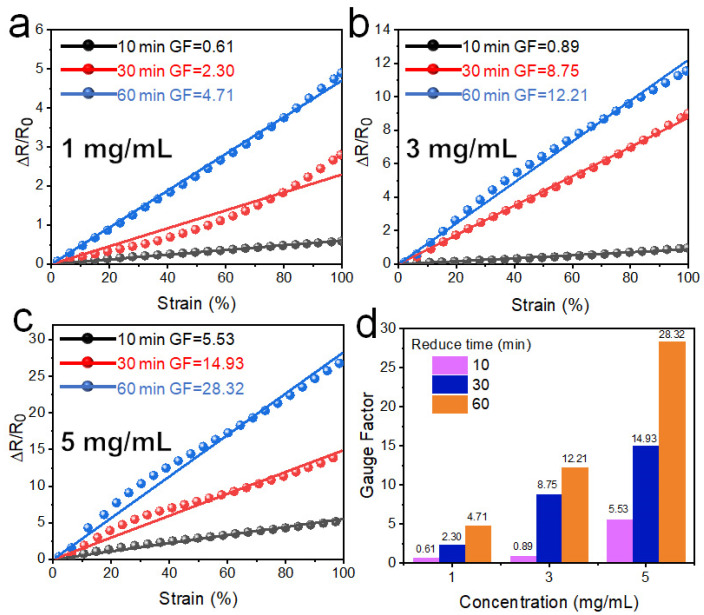
Strain-sensing curves in different reduction time at a concentrate of (**a**) 1 mg mL^−1^, (**b**) 3 mg mL^−1^, (**c**) 5 mg mL^−1^. (**d**) Statistical bar chart of GF in various concentrates and reduction time.

Figure 11 shows the relative resistance change as a function of the strain of WG@R series samples stretching along the direction of the secondary wrinkles, where GF is significantly higher than that of the primary wrinkles. Figure 10 shows that stretching in this direction separates the connections formed by secondary wrinkles, and this separation significantly reduces and prolongs the conductive path, resulting in a large resistance. In samples with the same GO concentrations, longer reduction time leads to greater GF; meanwhile, at the same reduction time, the higher the GO concentration was, the greater the GF was. This is contrary to the square resistance trend of the sample: the higher the square resistance, the lower the strain coefficient. Thus, this conclusion is contrary to the law of stretching along primary fold because it has a different response mechanism.

**Figure 12:**
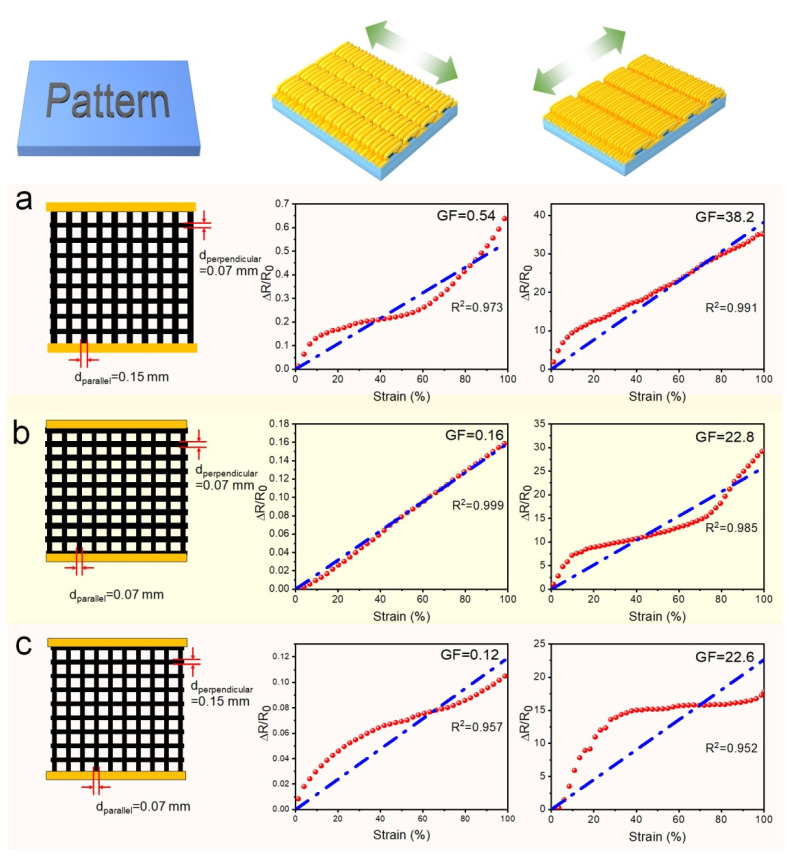
Strain-sensing properties along the directions of first- and second-grade wrinkles in PWG@R-5-60 of different patterns: (**a**) grid lines (bold lines parallel to stretching direction), (**b**) grid lines (normal), and (**c**) grid lines (bold lines perpendicular to stretching direction).

As shown in Figure 8, Figure 9, Figure 10 and Figure 11, WG@R has a unique anisotropy resulting from the two-stage shrinking. Based on the strain-sensitive characteristics in different strain directions, it can be used as a strain sensor and a strain-insensitive constant resistor, respectively. To combine the advantages of patterns and microstructures, some macro patterns were designed on this basis to achieve higher strain response and more stable resistance values. The strain coefficient of WG@R-5-60 was as high as 28.32 in the tensile direction of the secondary fold, and only 0.05 in the tensile direction of the primary fold; thus, it was selected as the patterned sample for further study. Moreover, to explore the theoretical basis of the influence of pattern on strain coefficient, the complex pattern is simplified into a grid along and perpendicular to the stretching direction, and the influence of horizontal and vertical lines is explored by the line thickness. The specific pattern design and strain curve are shown in Figure 12.

Figure 12a–c shows the grid pattern of the PWG-5-60 sample. We thickened the lines perpendicular to the stretching direction in grid (a) and the lines parallel to the stretching direction in grid (c). The thickening of lines perpendicular to the stretching direction was beneficial in reducing the GF, while the thickening of lines parallel to the stretching direction increased it. GF generated by the regular mesh (Figure 12b) is roughly equivalent to that of the sample without pattern. When the width of the grid lines perpendicular to the tensile direction is about twice that of the grid lines parallel to the tensile direction, the GF of the sample decreases from 0.15 to 0.12 in the tensile direction of the primary wrinkles. Meanwhile, when the width of the grid lines parallel to the tensile direction is twice that of the grid lines perpendicular to the tensile direction, the GF of the sample increases from 28.3 to 38.2.

**Figure 13:**
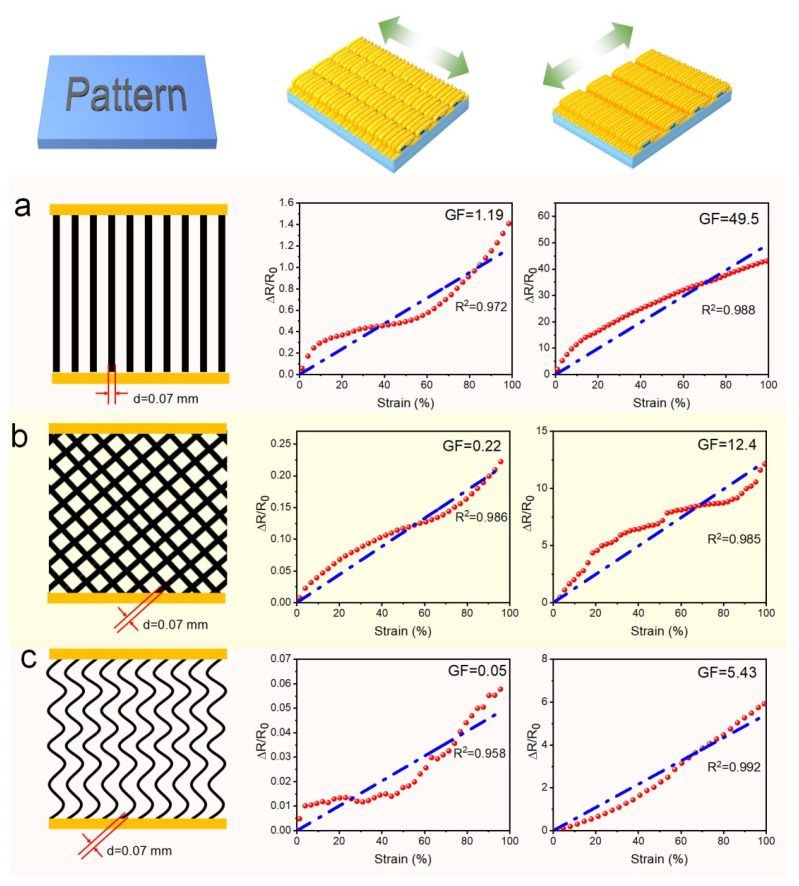
Strain-sensing properties along the directions of first- and second-grade wrinkles in PWG@R-5-60 of different patterns: (**a**) stripe, (**b**) grid lines (inclined), and (**c**) serpentine lines.

Based on the above experimental, patterns of striped lines, diagonal grids, and wavy lines were designed to obtain the required GF, as shown in Figure 13. The striped line design increased the degree of strain response, and the GF increased from 28.32 to 49.5 when stretched along the direction of second-order wrinkles. High GF is extremely rare for the non-crack strain sensor, and it is realized through the separation of surface connection and enhanced stripes. A 100% strain did not damage to the structure; thus, the assembly of the strain sensor has a high stability.

Moreover, the diagonal grid design increases the presence of perpendicular stripes, so it also shows GF reduction. However, owing to the existence of many edge outages and complex conductive paths, the effect is not significant. Meanwhile, the wavy line design has a great effect on reducing GF. Applying the wavy line design in the direction of primary wrinkles can reduce the GF from 0.15 to 0.05. Moreover, at most 5% variation in resistance within 100% strain is sufficient for most stable resistance devices. This strain-insensitive property is caused by the combination of structure and pattern, and so, the use of nanomaterials with better conductivity (such as RGO with better reducing degree [40], Ti_3_C_2_T_x_ MXene [41], or carbon nanotube (CNT) thin film [42]) can expand the application of this structure in the fields of stretchable electrodes and resistance stable conductivity.

We compared the performance of our sensors with those published in the literature [1,15,43,44,45,46,47,48]. The results are listed in Table S1. Compared with the literature, this work not only provides a strain sensor with a GF as high as 49.5 but also presents a method to tune the GF down to 0.05.

### 3.4. Wearable Applications of Stripe-Line PWG@R

**Figure 14:**
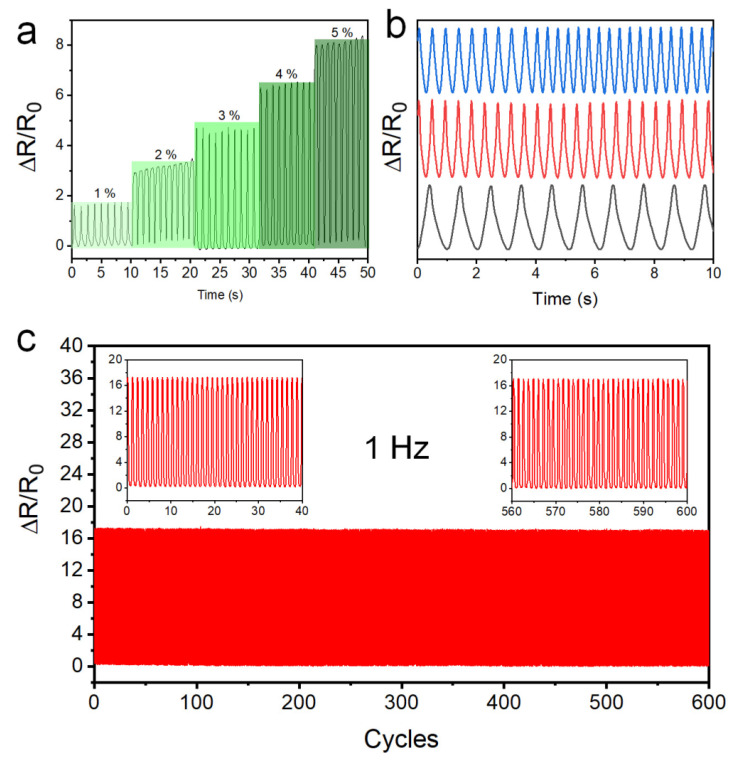
Strain-sensing properties and cyclable properties of stripe-line PWG@R-5-60-based strain sensors. Response at (**a**) various strains, (**b**) different frequencies, and (**c**) 600 cycles in 1 Hz at 15% strain (inset: the first 40 cycles (left) and the last 40 cycles (right)).

As shown in Figure 14a, the stripe-line PWG@R-5-60 strain sensor has a stable signal when different strains are applied. Its quick response and quick recovery are the basic characteristics of the strain sensor. Stability also includes its ability to cycle at different frequencies, as shown in Figure 14b, and repeatability at high frequencies, as shown in Figure 14c. During the 600 cycles of sensing process, the values of strain and resistance are stable. The uncertainty of the resistance measurement can be evaluated from the background blank testing of the measurement system. As shown in Figure S1, the variation of ∆R/R_0_ is as low as ±0.005%. The uncertainty of the strain measurement is under the error range of electronic mechanic testing machine (STD5000), ±0.1% (±0.01 mm).

**Figure 15:**
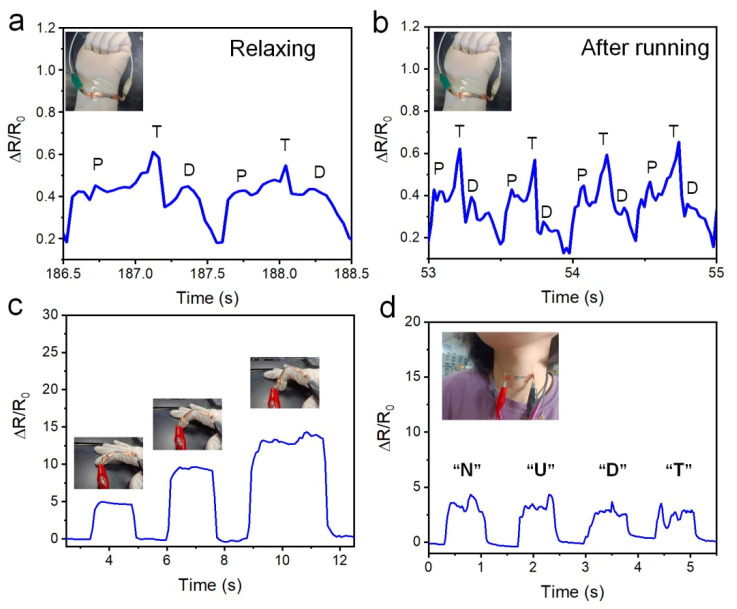
Demonstration of stripe-line PWG@R-5-60-based strain sensors detecting various human signals. (**a**) Wrist pulse in relaxation, (**b**) wrist pulse after running, (**c**) the bending of the finger, and (**d**) the pronunciation of different letters.

Figure 15 illustrates the potential of the stripe-line PWG@R-5-60 sample for human health detection. As shown in Figure 15a–b, the sensor can detect small signals of the wrist pulse and clearly distinguish the percussion peak (P), tide peak (T), and relaxation peak (D). The frequencies generated by rest and movement are easily identified.

The same sensor can also detect large movements, such as different finger bending angles, as shown in Figure 15c. Large and stable signal output can be generated under different finger bending conditions, which is of great significance for monitoring human movement. Similarly, this means that the strain sensor can be used as a motion detector on various joints in humans or robots. Figure 15d shows that the stripe-line PWG@R-5-60 strain sensor can tell the vibration generated by different letter pronunciations, indicating that this method has promising applications in speech recognition.

## 4. Conclusions

In this study, a functional electronic material, which is both customized and controllable, was successfully prepared by combining micro- and macro-structures. Wrinkling graphene films with regular structure and hierarchical orientation were obtained by two successive contractions of the pre-stretched substrate. When stretching along the direction of the primary wrinkles, the wrinkles widen and the distance increases, the conductive path does not change, so the resistance value changes little. Meanwhile, when strain is applied along the direction of the secondary wrinkles, the surface connection separates, shortening the conductive path and resulting in a large increase in resistance. To improve adjustability, macro patterns are designed for different applications. The results show that the enhancement of the vertical line increased the resistance change in the process of drawing, while the enhancement of the horizontal line decreased the resistance change. Guided by this theory, patterns of striped lines and wavy lines are applied to different drawing directions of PWG@R-5-60. The prepared stripe-line PWG@R-5-60 strain sensor has very high strain coefficient (GF = 49.5). Furthermore, the PWG@R-5-60 with a wavy line can be used as a constant resistance, and its resistance variation can be kept below 5% when the strain is up to 100%, which can meet the requirements of most electronic devices.

## Supplementary Materials

The following are available online at https://www.mdpi.com/article/10.3390/polym14142800/s1, Figure S1: Background blank testing of the resistance measurement system.; Table S1: Performance comparison of the strain sensors.
