# Supplementary material for: Hierarchical Wrinkles for Tunable Strain Sensing Based on Programmable, Anisotropic, and Patterned Graphene Hybrids
Source: Polymers (Basel). 2022 Jul 9;14(14):2800. doi: 10.3390/polym14142800 (PMC9322441; doi:10.3390/polym14142800)
Supplement: Supplementary file 1 [file polymers-14-02800-s001.zip › polymers-1777402-supplementary.pdf]

polymers

MDPI

Supplementary Materials

Hierarchical Wrinkles for Tunable Strain Sensing Based on Programmable, Anisotropic and Patterned Graphene Hybrids

Zengyong Chu [1,*], Guochen Li [1], Xiaofeng Gong [1], Zhenkai Zhao [1], Yinlong Tan [1] and Zhenhua Jiang [1]

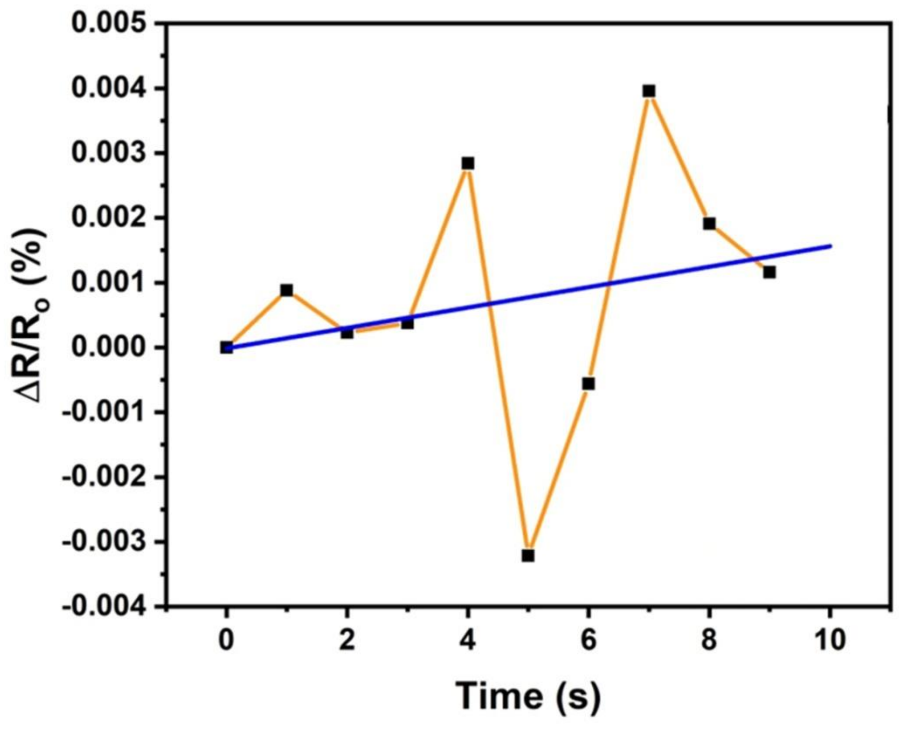

Figure S1. Background blank testing of the resistance measurement system.

Table S1. Performance comparison of the strain sensors

No.	Samples	GF	Strain range	Ref.
1	MWCNTs/PDMS	1140	0-8.75%	[1]
2	CB/PMSCSS	649	0-0.14%	[2]
3	RGO/Rubber	48.7	0-45%	[3]
4	Graphene	42.2	0-20%	[4]
5	PDMS/AgNW/PDMS	2-14	0-70%	[5]
6	CNTs	0.99	0-100%	[6]
7	RGO@Fiber	8.8	0-105%	[7]
8	RGO@nylon fabric	18.5	0-40.6	[8]
9	PWG@R-1	49.5	0-100%	This work
10	PWG@R-2	0.05	0-100%	This work

References

1. Nie, B.; Li, X.; Shao, J.; Li, X.; Tian, H.; Wang, D.; Zhang, Q.; Lu, B., Flexible and Transparent Strain Sensors with Embedded Multiwalled-Carbon-Nanotubes Meshes. *ACS Appl. Mater. Interfaces* 2017, 9, 40681−40689.
2. Song, H.; Zhang, J.; Chen, D.; Wang, K.; Niu, S.; Han, Z.; Ren, L., Superfast and High-Sensitivity Printable Strain Sensors with Bioinspired Micron-Scale Cracks. *Nanoscale* 2017, 9, 1166−1173.
3. Song, J.; Tan, Y.; Chu, Z.; Xiao, M.; Li, G.; Jiang, Z.; Wang, J.; Hu, T., Hierarchical Reduced Graphene Oxide Ridges for Stretchable, Wearable, and Washable Strain Sensors. *ACS Appl. Mater. Interfaces* 2019, 11 (1), 1283-1293.
4. Amjadi, M.; Pichitpajongkit, A.; Lee, S.; Ryu, S.; Park, I., Highly Stretchable and Sensitive Strain Sensor Based on Silver Nan-owireElastomer Nanocomposite. *ACS Nano* 2014, 8, 5154−5163.
5. Chun, S.; Choi, Y.; Park, W., All-graphene strain sensor on soft substrate. *Carbon* 2017, 116, 753-759.
6. Cohen, D. J.; Mitra, D.; Peterson, K.; Maharbiz, M. M. A Highly Elastic, Capacitive Strain Gauge Based on Percolating Nano-tube Networks. *Nano Lett.* 2012, 12, 1821−1825.
7. Mi, Q.; Wang, Q.; Zang, S.; Mao, G.; Zhang, J.; Ren, X. RGOCoated Elastic Fibres as Wearable Strain Sensors for Full-Scale Detection of Human Motions. *Smart Mater. Struct.* 2018, 27, 015014.
8. Lee, H.; Glasper, M. J.; Li, X.; Nychka, J. A.; Batcheller, J.; Chung,H. J.; Chen, Y. Preparation of Fabric Strain Sensor Based on Graphene for Human Motion Monitoring. *J. Mater. Sci.* 2018, 53, 9026−9033.
